# Demographic characteristics of women that use traditional birth attendants in Bongo District, Ghana

**DOI:** 10.18332/ejm/114884

**Published:** 2020-01-10

**Authors:** Prosper A. Adongo, Raymond A. Atanga, Vida N. Yakong

**Affiliations:** 1 Department of Community Health, University for Development Studies, Tamale, Ghana; 2 Department of Development Studies, University for Development Studies, Tamale, Ghana

**Keywords:** women, sociodemographic characteristics, traditional birth attendants, Bongo District

## Abstract

**INTRODUCTION:**

Over the last decade, the government of Ghana has implemented several interventions aimed at increasing access to skilled birth-care services from trained professionals. Despite these efforts, there is a wide gap between antenatal care attendance and skilled delivery attendance, particularly in rural areas. Evidence shows that many women in rural and deprived communities in Ghana rely on traditional birth attendant (TBA) delivery services. This has created a gap where antenatal attendance is high while skilled delivery is relatively low. The purpose of this study is to identify and analyse the sociodemographic characteristics of women who use the services of TBAs in Bongo District, Ghana.

**METHODS:**

Using a descriptive study design, a survey was conducted involving 330 mothers randomly selected from 1685 mothers who delivered at home by 2014 in Bongo District. The questionnaire for mothers who delivered at home by a TBA comprised 28 questions.

**RESULTS:**

The results show that women who used TBA were older, without formal education, married, predominantly farmers, married to spouses who were farmers without formal education. Most of the sampled women were co-currently covered by the national health insurance.

**CONCLUSIONS:**

This study describes the demographic characteristics of women who use a TBA. Therefore, ongoing efforts aimed at increasing access to and use of professional antenatal services should incorporate sociodemographic factors in the rural context.

## INTRODUCTION

Globally, traditional birth attendants (TBAs) handle 60–80% of all deliveries in the rural settings of developing countries^1^. Home births supervised by TBAs have long been associated with factors such as cultural norms and religious beliefs, cost, and accessibility of services. For instance, in Sub-Saharan Africa, the shortage of medical professionals for maternal health services compelled women to patronise maternal services provided by TBAs^1-4^.

The WHO^5^ defined a TBA as one (mainly a woman) who acquired her skills of delivering babies by herself, or working with other TBAs, and assists mothers during childbirth. TBAs are mostly initiated through internship with family and non-family TBAs and from dreams and revelations^6^. Their practice is associated with the exercise of spiritual and physical approaches involving the use of artefacts, herbs, as well as physical examination^6^.

Studies suggest that the role of TBAs is changing over time due to attempts by governments, health directorates and international organisations to limit, control, mainstream and in some cases outlaw their activities^7-10^. For instance, TBAs in some jurisdictions now play advocacy, sensitisation and referral roles instead of handling direct delivery cases^7-10^.

In Ghana, efforts have been made in recent times to limit the activities of TBAs in some areas to sensitisation and awareness-raising. Also, the introduction of free maternal health services for pregnant women and the introduction of the National Health Insurance (NHIS) in Ghana are expected to reduce the financial burden on pregnant women in order to increase access to skilled birth-care services provided by professionals^11^. However, recent studies in Sub-Saharan Africa and Ghana show that the practice of TBAs is still widespread and government policy of curtailing their services may not be successful as rural women still prefer the services of TBAs^12,13^ due to multiple factors militating against access to professional maternal care^14^. In rural settings, TBAs are known to offer affordable^15^ and accessible services as well as conduct delivery in an environment familiar to the woman^16,17^. Also, the preference for TBA services is due to their respect for the religious beliefs of clients^18^. In particular, women still use TBA services, especially those from rural and deprived communities^12^ such as the Bongo District in the northern part of Ghana. The situation has led to a wide gap between antenatal care attendance and skilled delivery attendance in Bongo District. For instance, in 2009, antenatal care attendance in Bongo District was 90.2% whereas skilled delivery performance was only 47.6%^19^. In 2010, antenatal care attendance increased to 96.5% whereas skilled delivery attendance increased marginally to 54.6%^19^. What one can infer from this high antenatal coverage and low skilled attendance at delivery is that most of the women attended antenatal care, but when it came to delivery, they delivered at home, probably through a TBA (relatives or friends). This implies that TBAs continue to play an important role in deliveries at home, especially in rural areas.

To date, there is a paucity of literature concerning the use of TBA services in the Bongo District of Ghana. This study, therefore, assesses the sociodemographic characteristics of women that influence their use of TBA services from the perspectives of women from Bongo District. The paper argues that the sociodemographic characteristics of women are important in explaining mothers’ choice of antenatal services. Consequently, this study seeks to widen the debate about the use of TBAs in Ghana.

## METHODS

### Study area

Bongo District was selected for the study because it recorded the lowest performance of skilled delivery services from 2008 to 2010 in the Upper East Region. This situation led to reported cases of four maternal deaths in 2008, five in 2009 and three in 2010^20^. The district lies between longitudes 0.45'W and latitudes 10°50'N to 11°9'N, with an area of 459.5 km^2^. It is located within the onchocerciasis-free zone of Ghana. Bongo District is bounded to the North and East by Burkina Faso, to the West by Kassena-Nankana Municipality and Kassena-Nankana West District and to the South by Bolgatanga Municipality.

The population of the district is about 84545, with 40084 males and 44461 females^21^. The district is predominantly rural, with over 80% of the inhabitants living in areas classified as rural. It is fairly homogeneous with the Boosi and Gurusi ethnic groups forming about 80% of the population^21^.

The modern healthcare system of the Bongo District is consists of one hospital in Bongo, the District capital, four health centres, thirteen completed Community-based Health Planning and Services (CHPS), sixty-two outreach stations, ten feeding/nutrition centres and one rehabilitation centre. The District Health Management Team (DHMT) headed by District Director of Health Services (DDHS) superintends the District Health Administration. The DHMT is supported by sub-district health management team (SDHMT) in all the six sub-districts with twenty-three midwives. Increase awareness creation on health promotion and protection was undertaken by community health nurses, community health officers, midwives and the Public Health Unit of the hospital, at the various health facilities, outreach points, schools and community durbars. Topics include environmental and personal hygiene, hand washing, good nutrition, seeking early treatment, know your HIV status, childcare, early initiation of antenatal care to women, free registration of pregnant women with NHIS, free maternity services for women, and many others.

### Material and methods

The study adopted a cross-sectional descriptive design. Descriptive research design helps assist the researcher in exploring and explaining a situation in its natural environment. Additionally, it permits the researcher to describe the characteristics of persons, situations and the occurrence of phenomena^22^. According to Glass and Stanley^23^ descriptive design is used when the researcher intends to accurately show the status of one or more variables, or to specify a phenomenon of human experience. The design has been adopted in this study in order to answer the questions: ‘what exists?’ or ‘how do the phenomena appear?’. The design is flexible and allows the researcher to choose quantitative or qualitative methods to answer the questions^24^. The descriptive study design was relevant to the study as the main objective was to identify factors influencing the use of TBAs.

### Sample size and sampling

The study populations included women who gave birth, within one year before the survey, in the study area. Out of a total of 1685 mothers who delivered at home by 2014, a sample of 330 was derived using the Yamane^24^ formula (n=N/[1+N×e^2^], where n = sample size, and e is the acceptable sampling error in a population N) at 95% confidence level (CI) and 5% margin of error.

### Sampling and sampling technique

A simple random sampling technique (lottery method with replacement)^25^ was employed to select 330 mothers who delivered at home by TBA in Bongo District for the study sample. The sample frame was obtained from the names and addresses of mothers available in child welfare clinics’ registers.

### Study instrument

Data were gathered from participants with the use of both closed and open-ended interview questions. Questionnaires for mothers who delivered at home by a traditional birth attendant (TBA) were administered.

### The Questionnaire

The questionnaire for mothers who delivered at home by TBA comprised 28 questions on demographic characteristics of the respondents. This set of questions was mainly close-ended. The questionnaire was pre-tested on 25 mothers attending a child welfare clinic in Bongo District hospital. Each mother was interviewed after the purpose of the study was explained. The purpose of pre-testing the instrument was to obtain clarity and to find out its appropriateness to the main study. There were no difficulties encountered with the questionnaire. However, the pre-test helped to modify some questions.

Data quality control was guaranteed throughout the process of data collection, coding, entry and analysis. Training exercises were conducted for all data collectors, and their work in the field supervised strictly for adherence. Supervision of data collectors included observation of how they were administering questions. Codes were assigned to questions in the questionnaires for easy detection and correction of errors. The completed questionnaires were checked for completeness by data collectors daily. Consequently, problems encountered were discussed among the survey team and addressed immediately.

Ethics constitute values that determine the degree to which research procedures adhere to professional, legal and social requirements about the study^26^. In order to satisfy the ethical requirement of the study, a research proposal was submitted by the researchers to the School of Allied Health Sciences, University for Development Studies for appraisal and approval. Following the approval by the University, further approvals were sought from the Regional Health Directorate of Upper East Region and Bongo District Health Directorate to use the district as the setting for the study.

At the community level, verbal informed consent was sought from participants to conduct interviews. Each respondent was made aware that participation in the study was optional – a respondent had the right to decide whether to participate in the study or not, and to withdraw from the study at any time if she so wished. Respondents were assured that information they provided would remain confidential and their identity kept anonymous. Consequently, respondents were asked to desist from writing their names on the questionnaire. Finally, each questionnaire was accompanied by a cover letter that explained the aims of the study as well as the rights and obligations of the research participant as well as the researcher. The research assistants used the local dialect to facilitate understanding and to seek participation, for those participants who could not read and write English.

### Data analysis

The completed questionnaires were checked for completeness and consistency of responses. The Statistical Package for Social Sciences (SPSS) computer software package (Version 16.0) was the tool used to analyse the data, organised mainly into frequency tables. Reasons that accounted for the use of and challenges associated with using a TBA were computed and presented in tables in a ranked order. Chi-squared test by cross-tabulation was also employed to measure how utilisation of TBA services relate to sociodemographic variables and other service characteristics. The study considered p-values <0.05 as significant.

## RESULTS

The study identified the demographic characteristics of women that reported use of TBA, shown in Table 1. Maternal age was significantly (p<0.001) associated with use of TBA, 180 (54.5%) of the respondents were aged 30–37 years while 90 (30.0%) were aged 20–29 years, indicating that the older women prefer to deliver more at home with a TBA. The educational level of mothers also influenced (p<0.001) the choice of TBA for delivery. Those with no formal education (61.2%) used the services of a TBA, followed by women who had basic level education (32.7%). Among the secondary and tertiary educational levels, 5.5% and 0.6% of respondents used a TBA, respectively. Religious affiliation also differed among those who chose a TBA for delivery. The data indicate that Traditionalists (53.9%) and Christians (28.5%) use TBA more than Muslims (17.6 %).

**Table 1:** Demographic characteristics of participants that use a traditional birth assistant (TBA) in Ghana (N=330)

*Variables*	*Categories*	*TBA n (%)*	*p*^1^
**Age** (years)	13–19	12 (3.6)	<0.001
	20–29	99 (30.0)	
	30–37	180 (54.5)	
	≥38	39 (11.8)	
**Maternal education**	No formal	202 (61.2)	<0.001
	Basic	108 (32.7)	
	Secondary	18 (5.5)	
	Tertiary	2 (0.6)	
**Religion**	Christian	94 (28.4)	<0.001
	Islamic	58 (17.6)	
	Traditional	178 (53.9)	
**Marital status**	Married	273 (82.7)	<0.001
	Single	22 (6.7)	
	Widowed	18 (5.5)	
	Co-habitation	12 (3.6)	
	Separated	4 (1.2)	
	Divorced	1 (0.3)	
**Maternal occupation**	Housewife	34 (10.3)	<0.001
	Civil servant	17 (5.2)	
	Farmer	230 (69.7)	
	Trader	30 (9.1)	
	Hairdresser	19 (5.8)	
**Husbands education**	No formal	219 (66.4)	<0.001
	Basic	68 (20.6)	
	Secondary	20 (6.1)	
	Tertiary	10 (3.0)	
	Missing value	13 (3.9)	
**Husbands occupation**	Civil servant	20 (6.1)	<0.001
	Farmer	268 (81.2)	
	Trader	24 (7.3)	
	Hairdressing	0 (0)	
	Dress maker	33 (0.9)	
	Missing value	15 (4.5)	
**Number of deliveries**	1–2	105 (31.8)	<0.001
	3–4	178 (53.9)	
	5–6	40 (12.1)	
	≥7	7 (2.1)	
**Insurance status**	Card bearers	309 (93.6)	<0.001
	Non card bearers	21 (6.4)	

1 Based on chi-squared test.

Women’s occupation was also observed to be significantly associated (p<0.001) with TBA use (Table 1). Within our study sample, the majority of respondents were farmers 230 (69.7%) followed by housewives 34 (10.3%). Moreover, partner’s educational status was significantly associated (p<0.001) with the choice of a TBA for delivery. The majority of women, 219 (66.4%), whose husbands had no formal education, used a TBA more than those who had basic education, 68 (20.6%), secondary education, 20 (6.1%), and tertiary education, 10 (0.3%). The data show that women whose husbands had lower educational level had greater chances of using a TBA for delivery compared to women whose husbands had higher education.

Finally, the chi-squared test showed a statistically significant relationship (p<0.001) between membership of the health insurance scheme and use of a TBA.

## DISCUSSION

The results from this study give the characteristics of rural women in Ghana that rely on the services of TBAs, despite government efforts to increase access to trained maternal services. The patronage of TBAs is significantly influenced by the sociodemographic characteristics of women. The findings indicate that older women prefer to deliver at home by TBAs than younger women. Studies on the association of a woman’s age and utilisation of TBAs have produced mixed results. Mwifadhi et al.^27^ and Siajabu^28^ in Tanzania have revealed that older women are more likely to access the services of TBAs than younger women.

In contrast, using empirical evidence from Kenya, Carter^29^ has argued that the age of the mother does not affect her choice of delivery service. Another perspective is presented by Constanze and Rosemarie^30^ in Tanzania and Ogolla^31^ in Kenya that suggests that younger mothers use the services of TBAs more than older women. These divergent views about the relationship between age of a mother and the use of TBAs can be explained by several factors inter alia, cultural, attitude of service providers, the perception about the quality of services and financial constraints. For instance, a study in Nigeria revealed that teenage mothers lack the financial resources to access services from professionals and therefore rely more on relatives or TBA services, which are relatively cheaper^1^.

Similarly, in Sub-Saharan Africa, TBA services are embedded in the cultural and traditional norms of the people and are therefore more acceptable to rural women than professional services from trained midwives^2,7,8,13^. This finding has several implications for maternal health, safe motherhood, and delivery. It implies that current efforts by the government through the Ministry of Health to expand the services of trained midwives cannot be achieved without sensitising rural women about the advantages of accessing services from professionals.

The study also reveals the importance of education in the health-seeking behaviour of rural women. Majority of mothers without formal education access the services of TBAs. This finding is consistent with Mwinfadhi et al.^27^, Henry and Tukur^32^ in Tanzania and Seter et al.^33^ in Iraq, whose studies suggest that women without formal education are more likely to access the services of TBAs than women with a formal education. In contrast, the Siajabu study from Tanzania proves the opposite. Again, these differences in health-seeking behaviour can be attributed to cultural, financial, and other factors^28^.

It has been revealed in this study that, the marital status of women also affects their utilization of TBAs. While married women are more likely to access the services of health professionals, single women prefer to deliver at home under the care of TBAs. Siajabu^28^ and Ogolla^31^, report similar situations in Tanzania and Kenya, respectively. The occupation of rural women also significantly affects their use of health facilities. The findings show that farmers and housewives tend to use the services of TBAs more than traders, civil servants, and hairdressers. This situation may stem from the fact that farming is embedded in the culture of local people, and therefore relates to the activities of TBAs. This is further supported by the finding that the majority of women who practice Traditional Religion use the services of TBAs. The importance of religion as a determinant of maternal healthcare utilisation has been emphasised by Addai^34^, Bazant and Koenig^35^, and Carter^29^. For instance, Addai^34^ has revealed that Christian women, particularly Roman Catholics, are more likely to seek antenatal care and skilled delivery care compared to mothers of Islamic and Traditional religions.

Several studies have shown that health insurance affects the use of TBA services^36,37^. These studies suggest that women who are active card holders of the National Health Insurance Scheme (NHIS) are more likely to use professional medical care than those without insurance. Contrary to this view, this study revealed that the majority of rural women use the services of a TBA regardless of health insurance status. The results show that over 93 per cent of women who are on the National Health Insurance Scheme use the services of a TBA. This implies that the utilisation of TBAs goes beyond the issues of affordability of professional care as earlier studies suggest.

The results also show that the parity of a woman influences her choice of TBAs for delivery. Low parity women had a higher tendency to use the services of TBAs than the high parity women. This finding opposes the notion by Obeng^38^ who studied factors influencing the utilisation of skilled delivery services in the Ofinso South District in Ghana, by Adjei^39^ who assessed factors contributing to low utilisation of skilled delivery services in Ahafo-Ano South District in Ghana, Amankwa^37^ who assessed the determinants of skilled birth attendance in the city of Bolgatanga Ghana, as well as Mengsteab^40^ who studied skilled attendance in Zona in South Africa. Their findings show that low parity women prefer the services of skilled professionals. The finding, however, confirms Ogolla^31^ whose study had revealed that first-time mothers were five times more likely to give birth at home compared to those who had two or more children. Attitudinal differences have sometimes been cited to explain the higher utilisation of health services by young, primipara women compared to older, multipara women. For instance, in the Ogolla^31^ study in Kenya, the reason for the teenage girls delivering at home was due to lack of awareness of the date of delivery, and their failure to inform their parents about their pregnancy, among others.

### Limitations and strengths

This study is cross-sectional and may not be generalizable to the entire childbearing population. While the study did not include a non TBA birth group, it is not possible to compare the demographical characteristics of the population that used a TBA versus the population that did not. Nevertheless, this study gives the demographic characteristics of those who used a TBA in Ghana, and area in which there is limited information available.

## CONCLUSIONS

This study in Bongo District gives the characteristics of rural women in Ghana that rely on the services of a TBA, despite government efforts to increase access to trained maternal services. The use of a TBA in rural Ghana is influenced by the sociodemographic characteristics of women. Taking these factors into consideration, a singular focus on increasing the availability of skilled birth attendants in rural areas does not appear to be a practical strategy at the moment; a more holistic approach is needed to deal with the issue. Furthermore, phasing out TBA training will be detrimental at this time, when most communities are without health facilities. Also, policies targeting increases in skilled attendance must work with the local cultures to create incentives for women. Noting that TBAs are an important source of delivery care, policy makers need to make the best use of them while simultaneously planning for their replacement with skilled birth attendants, since women are patronizing their services in rural areas.
