# Supplementary material for: Demographic characteristics of women that use traditional birth attendants in Bongo District, Ghana
Source: Eur J Midwifery. 2020 Jan 10;4:1. doi: 10.18332/ejm/114884 (PMC7839126; doi:10.18332/ejm/114884)
Supplement: Supplementary file 1 [file EJM-4-1-s1.pdf]

APPENDIX

Table 1: Husbands education and the use of Traditional Birth Attendants

Variable	TBA(n= 330)	X^2	DF	p-Value
No Formal	219(66.4)			
Basic	68(20.6)			
Secondary	20(6.1)			
Tertiary	10(3.0)			
Missing Value	13(3.9)	352.8	3	0.000

Table 2: Husbands Occupation and the use of Traditional Birth Attendants

Variable	TBA (n= 330)	X^2	DF	p-Value
Civil Servant	20 (6.1)			
Farmer	268 (81.2)			
Trader	24 (7.3)			
Hairdressing	0 (0)			
Dress Maker	33 (0.9)			
Missing Value	15 (4.5)	840.6	4	0.000

Table 3: Number of deliveries and the use of Traditional Birth Attendants

Number of Deliveries	TBA (n= 330)	X^2	DF	p-Value
1 – 2	105 (31.8)			
3 – 4	178 (53.9)			
5 – 6	40 (12.1)			

7+	7 (2.1)	207.7	3	0.000

Table 4: NHIS status and the use of traditional birth Attendants

NHIS Status	TBA (n= 330)	X^2	DF	p-Value
Card Bearers	309 (93.6)			
Non Card Bearers	21 (6.4)	824.7	3	0.000

© 2020 Adongo P. A. et al.
